# Cucurbitacin E inhibits cellular proliferation and enhances the chemo-response in gastric cancer by suppressing AKt activation

**DOI:** 10.7150/jca.31303

**Published:** 2019-10-06

**Authors:** Wenzhang Si, Jia Lyu, Zhengchuang Liu, Chunyang Wang, Jingjing Huang, Liping Jiang, Tonghui Ma

**Affiliations:** 1 Department of General Surgery, Affiliated Hospital of Shaoxing University (Shaoxing Municipal Hospital), Shaoxing, Zhejiang Province, China.; 2 Department of Urology, Zhejiang Provincial People's Hospital, Hangzhou Medical College, Hangzhou, Zhejiang 310014, China; 3 Key Laboratory of Gastroenterology of Zhejiang Province, Zhejiang Provincial People's Hospital, Hangzhou Medical College, Hangzhou, Zhejiang Province 310014, China; 4 Genetron Health (Hangzhou) Medical Laboratory Co. Ltd, Hangzhou 310000, China

**Keywords:** gastric cancer, Cucurbitacin E, doxorubicin, AKt

## Abstract

**Background:** The incidence and mortality rate of gastric cancer has markedly declined over the past few decades, due to the progress and advances in the development of diagnostic and treatment regimens. However, there is still a large portion of patients who are first diagnosed during the advanced stage of gastric cancer when chemotherapy is needed. Unfortunately, resistance to chemotherapeutic agents is the most frequent occurrence during treatment, which indicates a need for the discovery of novel therapeutic anticancer drugs. **Methods:** The tumor-suppression effect of eight different cucurbitacins was evaluated in gastric cancer cell lines, and the Cucurbitacin E (CuE) showing the greatest effect was used in further studies to explore the mechanism and potential synergistic effect of Dox both *in vitro* and *in vivo*. **Results:** Compared with other cucurbitacins, CuE showed the greatest antiproliferative activity against the gastric cancer cell lines. Further investigations revealed that CuE suppressed the growth of gastric cancer cell lines through the induction of G2/M arrest and subsequent apoptosis by impairing AKt activation and reducing its expression in gastric cancer cells. Furthermore, our results indicate that CuE can significantly enhance the cytotoxicity of doxorubicin (Dox) both *in vitro* and *in vivo*. **Conclusion:** In summary, we present the first evidence of the efficacy of CuE for the inhibition of gastric cancer growth and the synergistic antitumorigenic effect of CuE and Dox, both *in vitro* and *in vivo*.

## Introduction

Gastric cancer is the fourth most prevalent cancer^1^. It is also reportedly the second most lethal cancer worldwide, despite significant progress in detection and therapeutic regimens over the past decade. There is still a significant proportion of patients who receive the first diagnosis during the advanced gastric cancer, and hence, their prognosis is always associated with a poor outcome. The 5-year survival rate of these patients is only approximately 31% in the United States (stage IA 94%, stage IB 88%, stage IIA 82%, stage IIB 68%, stage IIIA 54%, stage IIIB 36%, stage IIIC 18%)^2^-^4^. Patients diagnosed at the advanced stage had a 5-year relative survival rate of 30% for males and 32% for females during the period 2002-2003 in Shanghai, China^5^. In practice, chemotherapy is more frequently administered to advanced-stage patients who have greater chances of benefitting from the treatment^6^. However, the resistance that develops during chemotherapy is a major obstacle for successful gastric cancer treatment. Most metastatic gastric cancers eventually develop resistance to chemotherapy, ultimately resulting in poor response rates^7^. Therefore, understanding the mechanisms by which tumors develop resistance to various agents is the key to identifying new genetic targets and combination therapies to fight resistance.

Recently, natural cucurbitacin compounds, which are tetracycline triterpene compounds derived from plants of the Cucurbitaceae family, have been shown to have effective pharmacological properties, such as anti-inflammatory and anticancer properties^8^-^12^. Cucurbitacin E (CuE), a cucurbitacin, has been shown to have remarkable potential in suppressing the proliferation of multiple cancer cell types^13^. Feng et al. demonstrated that CuE inhibits cancer cell progression via the inhibition of Wnt/β-catenin signaling, and Wang's results suggest that CuE suppresses cytokine expression in human Jurkat T cells by inhibiting the activation of NF-κB^13^, ^14^. The anticancer effects of cucurbitacins on diverse tumor types, such as breast cancer, neuroblastoma, lung cancer, endometrial cancer, and hepatocellular carcinoma, have been well studied. However, the efficacy of cucurbitacins for the suppression of gastric cancer growth has not been explored thus far^15^-^20^.

The present study sought to assess the antitumorigenic effects of different cucurbitacins on gastric cancer cells. Thereafter, we selected CuE, which was the most effective against gastric cancer cells for further study. In addition, CuE was administered to the xenografts to evaluate the antitumor activity *in vivo*. Then, we compared the tumor-suppressing efficacy of CuE alone and in combination with dox and investigated its potential anticancer mechanism. This study may be of significant functional relevance for exploration of cancer cell resistance to chemotherapeutic agents.

## Methods and materials

### Cell lines and compounds used

Human gastric cancer cell lines (NCI-N87, BGC-823, SNU-16, SGC-7901, and MGC-803) were purchased from the American Type Culture Collection (ATCC). Cucurbitacin A, B, D, S, IIa, and IIb (CuA, CuB, CuD, CuS, CuIIa and CuIIb) were obtained from ChemFaces (Wuhan, China). Cucurbitacin E and I (CuE and CuI) were from Sigma Aldrich (St. Louis, MO). The Cucurbitacins were dissolved using dimethyl sulfoxide (DMSO). Antibodies of PTEN, AKt, P-AKt and GADPH were procured from Cell Signaling Technology (Danvers, MA).

### Cell culture and treatments performed

Human gastric cancer cell lines (NCI-N87, BGC-823, SNU-16, SGC-7901, and MGC-803) were passaged in RPMI-1640 medium supplemented with 10% FBS (Invitrogen), 50 U/ml penicillin and 50 µg/ml streptomycin, at a temperature of 37°C in the presence of 5% CO_2_. Cellular growth was monitored using an inverted microscope. At 70-80% confluence, the cells were passaged using 0.25% trypsin. The cells were passaged every 3 to 4 days, and those in the logarithmic growth phase were used in experiments.

### Cellular viability

The efficacy of the indicated compounds on gastric cancer proliferation was evaluated using a Cell Counting Kit-8 (CCK8, Sigma, St. Louis, MO). In brief, cells in the logarithmic growth phase were left to adhere on 96-well microplates at a density of 1×10^3^ cells/ml in 200 μl of media containing 10% FBS. Twenty-four hours later, these cells were exposed to 10 µM solutions of different cucurbitacins or DMSO for 48 h, followed by the addition of 10 µl of CCK8. The absorbance at 450 nm was detected postincubation at 37ºC for 4 h, using a microplate reader (Bio-Tek, USA). The results were analyzed using GraphPad Prism 5, and we multiplied the ratio of absorbance of drug-treated cells with that of untreated cells and multiplied the value by 100 to obtain the percentage of cell viability.

### Apoptosis assay

Apoptosis was analyzed using a double-staining apoptosis detection kit with Annexin V-FITC and Propidium Iodide (PI) (eBioscience, San Diego, CA, USA). In brief, the NCI-N87 cells were treated with CuE (100 nM) or DMSO for 24 h. Cells were collected using 0.25% trypsin and centrifuged at 300×g for 5 min at room temperature, followed by washing twice in PBS. The cells were resuspended in 100 μl of an appropriate binding buffer and stained using Annexin V/PI. The cells were enumerated using BD FACSDiva software (Becton Dickinson) and the BD FACS Canto II system flow cytometry platform.

### Cell cycle analysis

The NCI-N87 cells were treated with CuE (100 nM) or DMSO for 24 h. A total of 5×10^5^ cells were harvested, washed twice with PBS, and then fixed overnight with 70% methanol at 4°C.The fixed cells were incubated with PI at 37°C for 30 min in the dark and then analyzed using a flow cytometer (BD FACS Canto II, Becton Dickinson).

### Western blotting

The NCI-N87 cells were treated with CuE (25 nM, 50 nM, 100 nM or 200 nM) or DMSO for 48 h. Cellular lysis was carried out in lysis buffer containing protease inhibitor cocktail Z (Roche Applied Science, Germany). Equal amounts of whole protein extracts were resolved using SDS-PAGE and blotted onto PVDF membranes, which were then incubated with diluted primary antibodies and secondary antibodies. The analysis was performed using an ECL western blotting analysis system (Bio-Rad, CA, USA). ImageJ software was used for densitometric analyses.

### *In vivo* xenografts

Female BALB/c nude mice (SLAC Laboratory Anim al, Shanghai, China) were housed at our institution and all animal experiments were conducted in accordance with institutional guidelines. Female BALB/c nude mice (4-5 weeks old) were subcutaneously injected with 1×10^7^ NCI-N87 cells (suspended in 100 μl of PBS with 50% Matrigel (BD Biosciences)) to establish tumors. We used calipers to measure tumor dimensions. The formula: length × width × height × 0.5236 was used for tumor volume enumeration^21^. Two weeks later, the tumor-bearing mice were segregated into four groups of 6 per group, and all groups were administered an intraperitoneal injection of either (i) 0.15 ml of PBS every 3 days, (ii) 0.30 mg/kg of CuE every 3 days, (iii) 2 mg/kg of Dox every 3 days, or (iv) 0.30 mg/kg of CuE and 2 mg/kg of Dox every 3 days^22^. We used the formula volume = width^2^ × length/2 to calculate the tumor volume (in mm^3^), and plotted a tumor growth curve. The tumors were monitored until the mice were sacrificed.

### Statistical analysis

We performed all experiments in triplicate, unless otherwise noted, and the data are presented as the mean ±S.D. GraphPad Prism software was used for calculations, and a p value of less than 0.05 was considered indicative of statistical significance.

## Results

### Cucurbitacins have a cytotoxic effect on cultured gastric cancer cells

**Figure 1:**
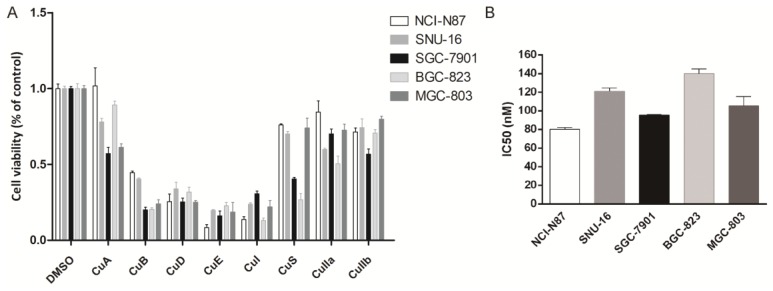
** CuE effectively inhibits gastric cell proliferation.** Cells (NCI-N87, SNU-16, MGC-803, SGC-7901, and BGC-823) were treated with various cucurbitacins as indicated. **(A)** Cell viability was estimated using CCK8 assays. **(B)** Cells were treated with different doses of CuE (from 0 nM to 300 nM), analyzed using the CCK8 assay, and the IC_50_ was measured.

Eight cucurbitacins (CuA, CuB, CuD, CuE, CuI, CuS, CuIIa, CuIIb) were incubated with five gastric cancer cell lines (NCI-N87, BGC-823, SNU-16, SGC-7901, and MGC-803) at a concentration of 10 µM for 48 h , to study the toxicity of cucurbitacins on gastric cancer cells. We measured cellular viability using a CCK8 assay. All cucurbitacins exhibited antitumor efficacy on gastric cancer growth, compared with that of DMSO. The antiproliferative effect of CuB, CuD, CuE and CuI was found to be more pronounced than that of the other cucurbitacins, while CuE showed the highest potential out of all the cucurbitacins, by killing more than 70% of cells (Fig. 1A). In order to further investigate how CuE affects gastric cancer, all five cell lines mentioned above were treated with varying concentrations (from 0 nM to 300 nM) of CuE. CuE exhibited dose dependent cytotoxicity on gastric cancer cells and its IC_50_ (half maximal inhibitory concentration) ranged from 80 nM to 130 nM (Fig. 1B).

**Figure 2:**
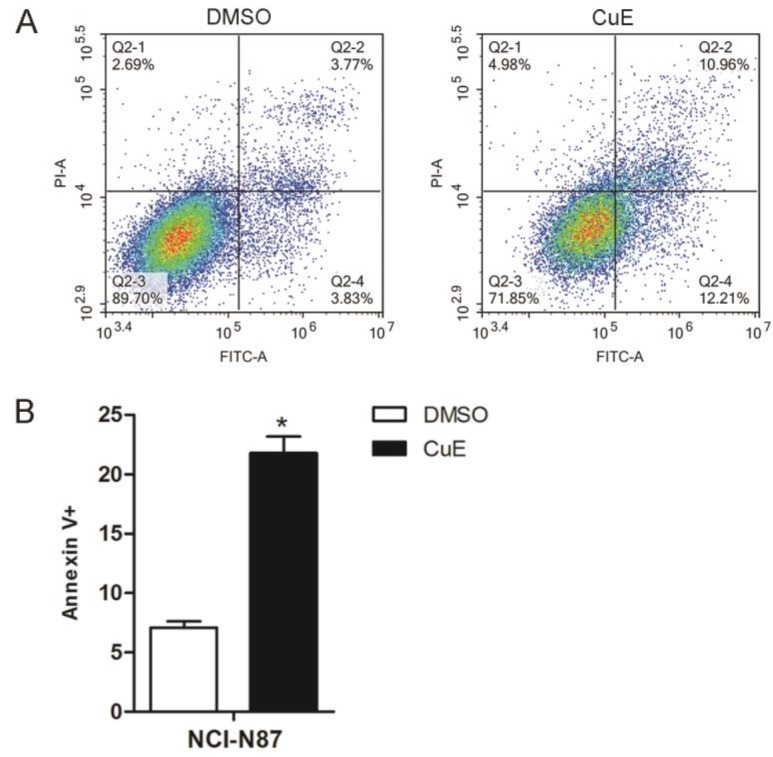
** CuE induces apoptosis of NCI-N87 cells.** NCI-N87 cells were exposed to CuE (100 nM) or DMSO for 24 h, and apoptosis was assessed through Annexin V/PI staining. **(A)** The effects of CuE on apoptosis assessed using flow cytometry are presented. **(B)** The percentage of Annexin V+ cells, which represent apoptotic cells, is shown (p = 0.0014).

In order to further confirm that CuE induces apoptosis, the NCI-N87 cell line was treated with CuE (100 nM), and DMSO was used as the control. After 24 h of incubation, Annexin V/PI staining of the cells revealed a higher percentage of apoptotic cells upon CuE treatment than upon DMSO treatment (Fig. 2A and 2B).

### CuE causes changes in cell cycle distribution by suppressing the activation of AKt

**Figure 3:**
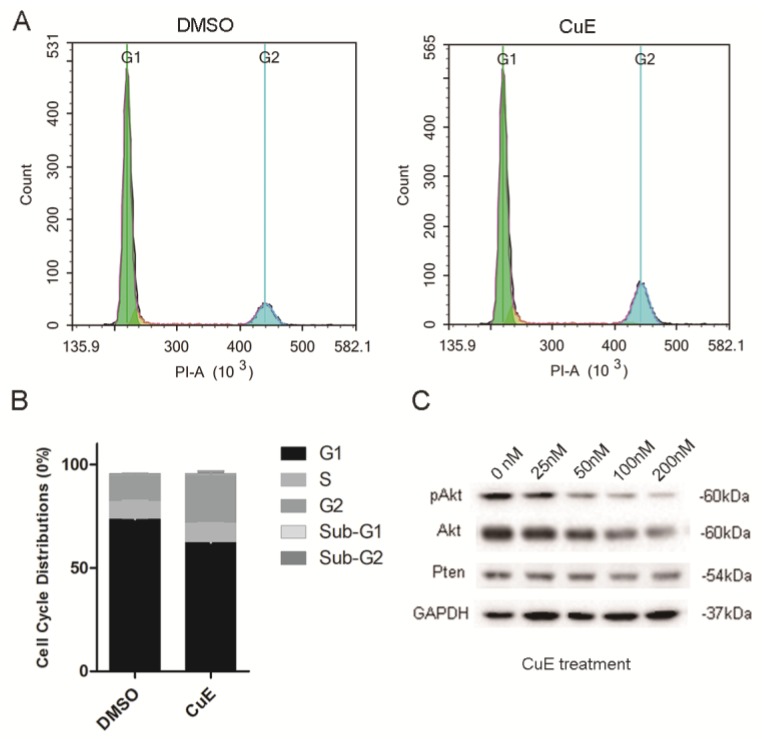
** CuE induces G2/M arrest and inhibits AKt activation of NCI-N87 cells. (A)** NCI-N87 cells were exposed to 100 nM CuE or DMSO for 24 h, followed by PI staining and cell cycle profiling using flow cytometric analysis. The horizontal and vertical axes represent the intensity of PI staining and cell counts, respectively. **(B)** Quantitative data of cell cycle distribution are shown in panel A. **(C)** NCI-N87 cells were cultured in the presence of DMSO or increasing concentrations of CuE (25 nM, 50 nM, 100 nM or 200 nM) for 48 h, and the cells were lysed for western blotting analysis.

The NCI-N87 cells were incubated with CuE (100 nM) or DMSO for 24 h to explore the inhibitory mechanism of CuE. Flow cytometric analysis of the cell cycle showed that CuE treatment caused G2/M arrest and significantly increased the proportion of cells that were at the G2/M phase (Fig. 3A and 3B).

**Figure 4:**
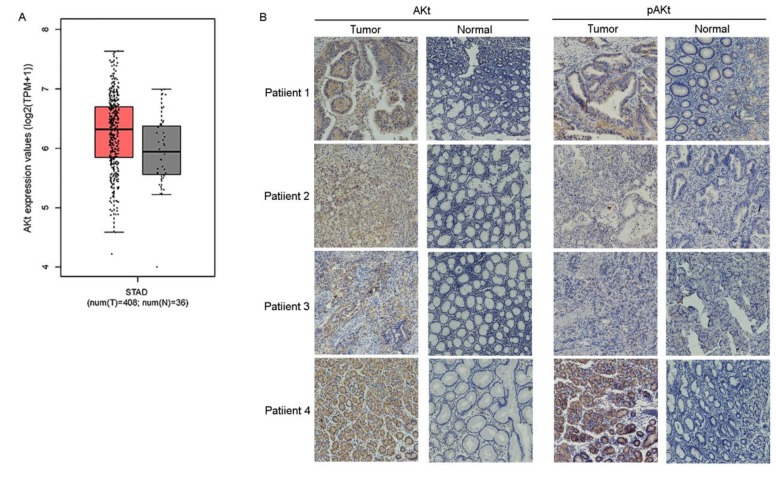
** AKt mRNA levels was higher in gastric tumors than in normal tissue in IHC assay.** Analysis of AKt1 mRNA levels in gastric cancer tumors and normal tissue from TCGA data **(A)** and immunohistochemical analysis of the expression levels of Akt and pAKt in 4 pairs of gastric cancer and normal tissue from cancer patients **(B)**.

Previous studies have suggested that preventing the activation of AKt induces G2/M arrest in diverse types of cancer cells, such as breast, lung and prostate cancer cells^23^-^25^. CuE has been suggested to inhibit the activation of AKt and enhance PTEN expression in lung cancer cells^26^. Therefore, the effect of CuE on AKt in gastric cancer cells was studied. After incubation with varying concentrations of CuE, we found a dose-dependent decrease in both pAKt and AKt levels, as shown by western blotting analysis. This observation implies that CuE suppresses the activation of AKt in gastric cancer cells in a dose-dependent manner, and inhibits the expression of AKt but shows no effect on PTEN (Fig. 3C). Then, we analyzed AKt mRNA levels in gastric cancer tumors and normal tissue using data from The Cancer Genome Atlas (TCGA) and found that AKt mRNA levels were higher in gastric tumors than in normal tissue (Fig. 4A). In addition, IHC detection of AKt and pAKt was performed on 4 pairs of gastric cancer and normal tissue specimens obtained from cancer patients. The relative protein expression intensity of AKt and pAKt was higher in gastric cancer tissues than in normal tissues (Fig. 4B).

### Suppression of AKt activation by CuE potentiates the cytotoxicity of Dox in NCI-N87 cells

**Figure 5:**
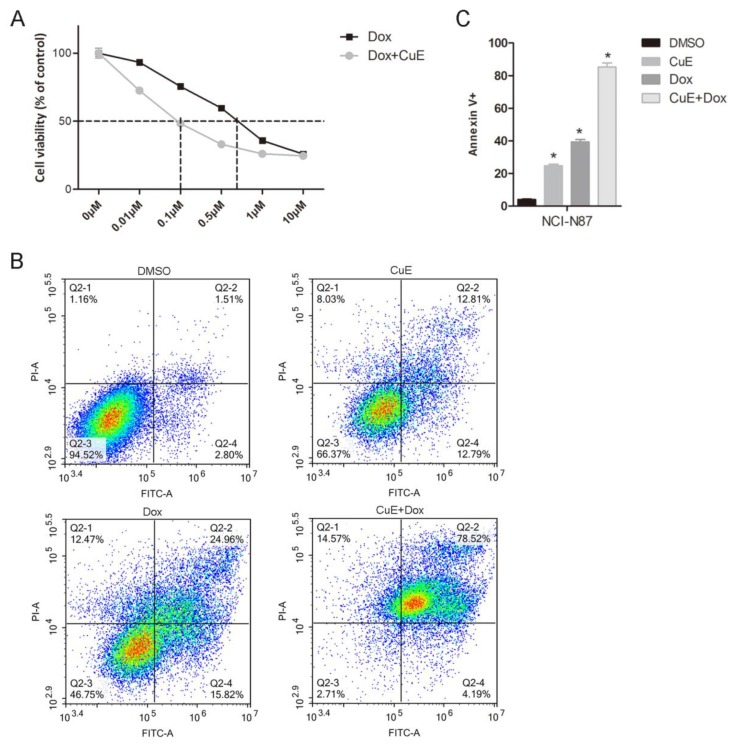
** The suppression of AKt activation by CuE potentiates Dox-induced cytotoxicity in NCI-N87 cells.** NCI-N87 cells were treated with Dox (0 μM, 0.01 μM, 0.1 μM, 0.5 μM, 1 μM or 10 μM) or a combination of CuE (60 nM) plus Dox (0 μM, 0.01 μM, 0.1 μM, 0.5 μM, 1 μM or 10 μM) for 48 h. **(A)** A CCK8 assay was used to measure cell viability. **(B)** NCI-N87 cells were treated with DMSO, Dox (500 nM), CuE (60 nM) or a combination of Dox (500 nM) plus CuE (60 nM) for 48 h, subjected to Annexin V/PI staining, and analyzed by flow cytometry. **(C)** The percentage of Annexin V-positive cells representing apoptotic cells is shown (CuE+Dox vs. Dox, p= 0.0044).

Recently, several studies have demonstrated the synergistic effects of AKt inhibitors in combination with Dox. Babichev found that inhibiting the PI3K/AKt/mTOR pathway could significantly enhance the effect of Dox on LMS cell lines, and the tumor volume of the group treated with the AKt inhibitor BEZ235 in combination with Dox decreased by 71% in comparison with that of the vehicle-treated group^27^. Hu and colleagues revealed that NVP-BKM120 (BKM120), a selective inhibitor of PI3K, also suppresses the activation of AKt and that it shows a strong synergistic antiproliferative effect in multidrug resistant (MDR) breast cancer cells, when combined with Dox^28^. Since CuE inhibits AKt activation, we explored whether CuE could enhance the cytotoxicity of Dox in gastric cancer cells. As expected, compared with Dox treatment alone, the combined CuE (60 nM) treatment significantly increased the sensitivity of NCI-N87 gastric cancer cells to Dox (IC50: 100 nM vs. 700 nM) (Fig. 5A). To further assess whether CuE/Dox-induces cell death is due to apoptosis, Annexin V/PI staining assay was performed and quantified by flow cytometry. As illustrated in Fig. 5B and 5C, compared to the Dox-only treated group, the CuE/Dox-treated group showed a significantly increased percentage of Annexin V-positive apoptotic cells (40% vs. 80%). These results demonstrate the potential of CuE to increase the cytotoxicity of Dox in NCI-N87 gastric cancer cells by promoting apoptosis.

### Synergistic growth inhibition of gastric cancer xenografts by CuE and Dox

**Figure 6:**
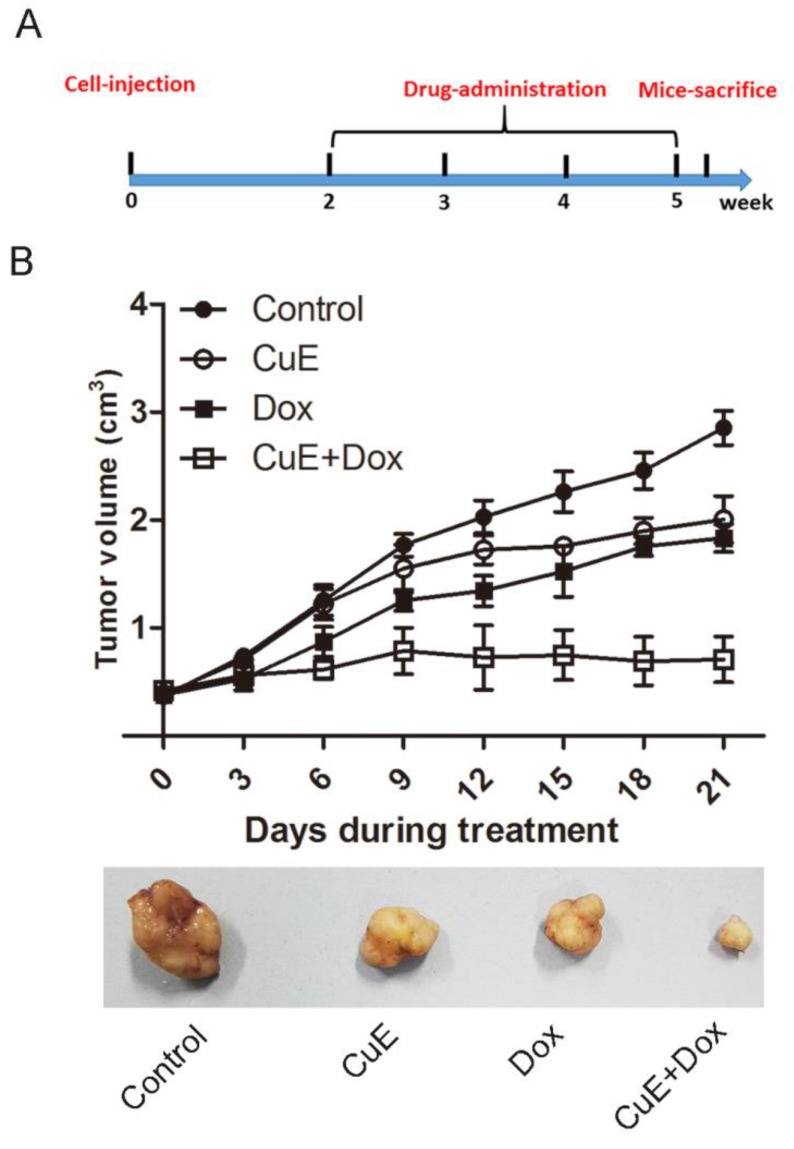
** The synergistic inhibition of gastric xenografts by CuE and Dox.** Four- to five-week-old female nude mice were injected on their right flank with 1×10^7^ NCI-N87 cells suspended in 100 µl of PBS with 50% Matrigel. One week later, the mice were randomly segregated into four treatment groups: control group (DMSO), CuE group (0.30 mg/kg, every 3 days), Dox group (2 mg/kg, every 3 days), CuE plus Dox group (0.30 mg/kg+2 mg/kg, every 3 days). **(A)** The experimental strategy is shown. **(B)** Tumor diameters were measured every 3 days, until the mice were sacrificed, and a representative tumor from each group is shown.

It has previously been observed that CuE inhibits the proliferation of gastric NCI-N87 cancer cells and that it enhances the sensitivity of NCI-N87 cells to Dox *in vitro*. We evaluated the effects of CuE and Dox monotherapy, as well as the combination therapy on the growth of NCI-N87 xenografts. BALB/c mice bearing NCI-N87 xenografts were arbitrarily divided into 4 groups and injected intraperitoneally with DMSO (placebo), CuE, Dox, or the combination of CuE and Dox for a period of 4 weeks (Fig. 6A). The animals were sacrificed, and tumor volumes were estimated 40 days post establishment of the xenografts. Consistent with the *in vitro* results, the combination group showed significantly lower volumes of xenograft tumors than the CuE and Dox monotherapy groups, which indicates that combination therapy is more effective in abrogating cellular proliferation than the vehicle or either monotherapy (Fig. 6B).

## Discussion

Although major advances have been made regarding gastric cancer treatments, patients with advanced stages of the disease still show a poor prognosis. Unfortunately, the OS of gastric cancer patients receiving systemic chemotherapy during the late stage is less than 1 year^29^. Chemoresistance in some forms of these cancers is still a significant challenge for the treatment of cancer and clearly requires more effective and safe regimens^30^.

Research concerning combinatorial chemotherapy has gradually gained attention from researchers in the field of gastric cancer research. Additionally, several recent reports have demonstrated the inhibitory effects of cucurbitacins on human cancer cells and tumor xenografts through the inhibition of STAT3 phosphorylation^31^, ^32^. Cucurbitacins are components of plant-based medicines used in Asian countries, such as China and India. Cucurbitacins are known for their antiproliferative activity on diverse forms of cancers^33^, ^34^.

This study investigated the effect of cucurbitacins on gastric cancer proliferation and elucidated the underlying mechanism of action. All eight cucurbitacins used in the study produced an antitumor effect on gastric cancer growth when used on the five included gastric cancer cell lines. Despite recent reports that CuI inhibits gastric cancer cell growth, interestingly, we found that CuE possesses greater inhibitory potential than CuI, the cause of which might be ascribed to diverse molecular mechanisms in different gastric cancer cell lines^35^.

We explored the inhibitory mechanisms of CuE on gastric cancer through the apoptosis and the cell cycle distribution of CuE- or DMSO-treated NCI-N87 cells. CuE treatment induced significant levels of apoptosis in comparison with the placebo treatment. Further investigations revealed that CuE induced G2/M arrest in NCI-N87 cells (Fig. 3A and 3B). We noted that previous work revealed the importance of the activation state of AKt in cellular apoptosis. Kim and colleagues demonstrated that activation of the PI3-K/AKt pathway reduces ASK1-induced apoptosis^36^. In contrast, Frias et al. found that AKt inhibitors (AKti-1/2 and A-443654) induce the apoptosis of chronic lymphocytic leukemia cells in a dose-dependent manner^37^. In addition to playing a key role in apoptosis, AKt is also required for the transition of cells into the G2/M phase^38^. Treatment of avian embryo retinas with a PI3K inhibitor (LY 294002) induced G2/M inhibition in late progenitors, which was mediated through the suppression of phospho-AKt^39^. Therefore, we evaluated the function of AKt in CuE-mediated inhibition of gastric cancer and found that CuE inhibits phospho-AKt in a dose-dependent manner. Interestingly, AKt levels also decreased along with the concomitant increase in the concentration of CuE, which was hitherto unknown. It has been previously reported that CuB, a member of the cucurbitacin family, upregulates PTEN expression and thereby affects the subsequent activation of AKt; we also found that CuE has little effect on PTEN expression (Fig. 3C).

Previous studies have suggested that the activation of PI3K/AKt signaling is frequently involved in the development of resistance to chemotherapy^40^, ^41^. Once activated, AKt activates multiple substrates and subsequent effectors to promote cell survival, proliferation and chemoresistance. Suppression of AKt activation by PI3K or AKt inhibitors has been suggested to be a valid approach for treating cancer and increasing the efficacy of chemotherapy^42^. In this study, we found that CuE treatment clearly inhibited AKt activation and boosted the cytotoxicity of chemotherapeutic agents such as Dox *in vitro* and *in vivo*, which indicates a clear synergistic effect with Dox in gastric cancer.

Chemotherapy is an important treatment option for advanced gastric cancers. However, the frequent occurrence of resistance to chemotherapy impairs the response rates of patients. Various efforts, such as combinatorial drug therapy, have been used to overcome chemoresistance. Our present study demonstrates the efficacy of CuE in different cancer cells of gastric origin. We have also identified, for the first time, the role CuE plays in the inhibition of gastric tumor growth by targeting the AKt signaling pathway. Furthermore, our results demonstrate that CuE displays the potential to enhance gastric cancer sensitivity to Dox-dependent treatment regimens. Our study provides a strong clinical rationale for the use of CuE as a novel pharmaceutical chemosensitizer for the treatment of gastric cancer.
